# Integrating mental health into primary care in Arkhangelsk County, Russia: the Pomor model in psychiatry

**DOI:** 10.1186/s13033-019-0271-1

**Published:** 2019-03-13

**Authors:** Grigory Rezvy, Elena Andreeva, Nadezhda Ryzhkova, Vera Yashkovich, Tore Sørlie

**Affiliations:** 1 Finnmark Hospital Trust, Kirkenes, Norway; 2 University of Tromsø-The Arctic University of Norway, Tromsø, Norway; 3 Northern State Medical University, Arkhangelsk, Russia; 4 Primorsky Central District Hospital, Arkhangelsk Oblast, Russia; 5 Arkhangelsk Psychoneurological Dispenser, Arkhangelsk, Russia; 6 Department of Mental Health and Substance Abuse, University Hospital of North Norway, Tromsø, Norway

**Keywords:** GPs, Information Communication Technology (ICT), Pomor model in psychiatry, Primary care, Specialised community mental health centre

## Abstract

**Background:**

Primary health care is still peripheral in the identification and treatment of mental health and substance use disorders in the Russian Federation. However, the development of primary health services has been given priority. A long-standing collaboration between Arkhangelsk County and northern Norway on mental health service development in Arkhangelsk has promoted the integration of mental health into primary care.

**Aim:**

To develop a model for mental health integration into primary care adapted to the conditions in Arkhangelsk County.

**Methods:**

(a) Situational assessment, (b) development of a model for systematic cooperation between GPs and specialists, (c) initial evaluation of the model, (d) implementation and dissemination of the model.

**Results:**

A local studies revealed major shortcomings in GPs’ diagnostic and treatment skills and in their collaboration with specialists in psychiatry. In order to promote better communication between GPs and specialists in this desolate and sparsely populated geographical area, an information communication technology (ICT)-based competence centre was established at a specialised community mental health centre in Arkhangelsk city (APND). Through a network including APND and involved primary health care centres, GPs gained access to specialists’ expertise when required in their work with psychiatric patients. GPs assess all patients’ mental health condition and treatment responsibility for patients in need of mental health care is divided between GPs and specialists according to problem severity. APND has the formal responsibility for ensuring that this collaboration with the health centres is established and practiced. Training in diagnostics and conversation skills ensures basic professional competence in the GPs. Initial evaluation showed that patients, GPs and specialists were satisfied with their experiences. The model is currently under implementation in 50% of the districts of the county.

**Conclusion:**

Our cooperation has led to the development and implementation of a model for mental health care integration into primary care in an area with major geographical distances. Further improvements will be based on systematic evaluation of experiences with the model.

## Background

The collapse of the Soviet Union in 1991 led to the introduction of a market economy associated with extensive economic and public health problems and a process of depopulation in the Russian Federation. During the period 1993–2010, the total population decrease was about 13 million people [1]. Non-communicable diseases such as high rates of mental illness, suicide, and alcohol abuse strongly contributed [2–4]. In a Russian cultural context, alcohol abuse may function more as escape from negative emotions and suicidality than in a Western Norwegian context [4], with negative implications for self-regulation, help-seeking behaviour and suicide rates.

Globally, mental disorders are leading causes of morbidity, with 12 months prevalence rates of about 25% in the general population [5]. The global burden of diseases attributable to mental and substance use disorders is correspondingly high [6]. Mediated through lifestyle habits related to smoking, eating habits, physical activity, and use of alcohol and drugs, mental health problems strongly influence premature mortality of the most common somatic disorders [7]. In a meta-analysis of studies of somatic morbidity in diverse mental and substance use disorders, the median years of potential life lost was 10 years [8]. These associations are strongest in serious mental disorders such as schizophrenia, with a weighted average of 14.5 years of potential life lost, more for men than for women [9].

In addition to preventive measures, the need for mental health care in a population requires that both primary and specialist health services have sufficient competence and capacity, and are cooperating well. However, depending upon political and professional priority setting, workforce and workload, psychiatric education, geographic distances, and how the cooperation between primary health care providers and specialists is organised, there is great variability between countries. In Norway, a model for organised cooperation between specialised community mental health centres (DPSs) and primary health services has been introduced in their respective geographical areas of responsibility [10]. The intention is that the DPSs should be active drivers of cooperation; but the model has been implemented to varying degrees, and in many areas, the DPSs function as regular reference agencies for primary care without further involvement in integrating mental health care into primary care.

Telemedicine aids are used to promote cooperation between primary health care providers and specialists in mental health care [11], and studies show that the use of information and communication technology (ICT) promotes easier patient access to health care providers and improved communication between caregivers and patients, especially where geographical distance poses a challenge [12].

In most Western countries, primary care general practitioners (GPs) play a central role in identifying and treating patients with mental health problems as well as in coordinating health care resources within municipalities and in cooperation with specialised services. During their lifetime, about 80% of the population of industrialised Western countries consult a GP, of whom between roughly 30 and 40% have significant psychiatric symptoms [13]. The fact that between 30 and 70% of these conditions remain undetected by GPs [14] underlines the need for adequate diagnostic training.

Over the last 10 years, steps have been taken to improve Russian primary health care, but there is still excessive specialisation within primary care, where only 16% of all physicians are district physicians or GPs, as compared with 30–50% in Western Europe [15]. Resources to support the system’s modernisation have been insufficient, and primary health care and local social services are still peripheral in the treatment of people with mental disorders. Large psychiatric hospitals, which mainly treat people with serious mental disorders, often with comorbid alcohol abuse disorders [16] are still cornerstones in mental health care. Alcohol- and substance abuse disorders are treated in the ‘narcology’ services, and are mostly separate from regular mental healthcare [17, 18]. In addition, psychiatrists at multispecialised polyclinics (MP), including district physicians, provide specialised and general outpatient services for populations residing in specific territories [17, 18]. In some cities (Arkhangelsk, Severodvinsk, Kotlas), psychiatric outpatient clinics or dispensaries (PDs) with psychiatrists, nurses, psychologists, and social workers, provide general psychiatric care. In peripheral district areas, small primary care practices (FAP) staffed by local nurses and feldshers (medical practitioners with less medical competence than a physician) are responsible for primary care. Ambulatory teams from the MPs give them support [16]. Solo GPs practice together with a nurse in some rural districts. Russia’s strong emphasis on specialised services relates to ideology and tradition [19], heavy mental health stigma, and a lack of economic resources [18].

In addition to the national ambition to strengthen primary care, the 1992 law of the Russian Federation on psychiatric care proclaiming the rights of individuals with mental health problems and that health professionals other than psychiatry specialists can diagnose and treat people with mental disorders [20] have stimulated initiatives to strengthen mental health care in primary care. In Sverdlovsk, a multi-component programme in primary care has contributed to sustainable training about common mental disorders being well integrated into the programme of health sector reforms [2]. Various factors such as a narrow bio-medical model mainly focusing on medical treatment, prognostic and therapeutic pessimism, hierarchical clinical decision-making limiting input from professionals other than psychiatrists, and an incorrect belief that treating mental health problems in primary care is not allowed, appeared to impede the integration of mental health care into the primary health service [21].

For the last 20 years, Russian and North Norwegian partners have collaborated on transcultural psychiatric research and mental service development in Arkhangelsk County. Comparative surveys of psychiatric health services [17], diagnostic practices [22], and acute psychiatric patients in Arkhangelsk and northern Norway have been conducted [4, 16], and there have been repeated reciprocal visits to both the primary- and specialist health services in both countries, resulting in good mutual knowledge of the quality and organisation of the services in both countries.

Since 2011, our cooperation has mainly been concentrated on the development and implementation of a model for systematic mental health care cooperation between primary care and specialist health services [23]. Arkhangelsk neuropsychiatric dispenser (APND), a primary health care centre close to Arkhangelsk, a group of Russian and Norwegian GPs, and the Russian–Norwegian project group have been involved.

In addition, central political decision-makers and representatives of the Northern State Medical University in Arkhangelsk (NSMU) have contributed. Meetings with the health minister in Arkhangelsk County and the role of the Russian project manager as his advisor in psychiatric matters, allow for a continuous dialogue with the minister throughout the process.

Relationship building, mutual understanding and cooperation have been facilitated by continuous good translation between languages and cultures. One project group member, (GR), speaks both languages fluently and has in-depth knowledge of the culture and psychiatry of both countries, and has played a central role in meetings and visits as a translator [24].

This article describes the development and implementation of a model for integrating mental health into primary care—the Pomor model in psychiatry in Arkhangelsk County located in northwest Russia.

## Methods

(a) Situation assessment, (b) development of a model for systematic cooperation between GPs and specialists, (c) initial evaluation of the model, and (d) implementation and dissemination of the Pomor model.

## Results

### Situation assessment

Since psychiatry in Russia has traditionally mainly relied on specialised services, psychiatric education has been modest both in the curriculum for medical students and in the specialisation for general/family medicine. As a result, the tradition of cooperation between primary and specialised care has historically been weak.

In 2011, a qualitative study was conducted in order to gain more knowledge about the GPs’ competence in psychiatry and their experiences of working with specialists on psychiatric patients [25]. To optimise variation in experiences and attitudes, participants (20 GPs and 11 psychiatrists) were selected to secure differences in gender, age, specialty, workplace, and duration of clinical experience. All participating GPs were in clinical positions at rural or urban local health centres in Arkhangelsk County. The participating psychiatrists were all recruited from the specialised community mental health centre located in Archangelsk city (APND) with different attitudes towards the idea of cooperation with GPs. Two researchers (GR and EA) who were familiar with explorative interview methods conducted three focus groups with GPs and two with specialists, using a prepared interview guide. Interviews were audiotaped and later transcribed verbatim. Four researchers (two psychiatrists and two GPs) independently assigned labels to what they considered the most important statements in the transcripts. They then discussed commonalities and discrepancies in the findings until a consensus was reached. The interviews were analysed consecutively in order to elaborate on important findings in prior interviews and to determine data saturation. For analysis, the computer program NVivo was used.

The findings indicated severe weaknesses in existing cooperation between GPs and psychiatrists. Although the GPs regularly met with patients with mental health problems, they had virtually no dialogue with specialists, even on patients who had previously received specialist treatment. The GPs rarely received feedback on patients they had referred to specialist treatment and the patients were often reluctant to provide GPs with specialists’ written reports. This pattern was confirmed by the specialists. The GPs considered their own ability to diagnose and treat people with mental health problems to be poor. Although they could often understand that patients had mental health problems, they had neither diagnostic expertise nor belief that they could help them. They had some guidelines on how to follow up patients after completing specialist treatment, but they expressed a wish to improve their treatment competence, and considered a more extensive collaboration with psychiatrists as the best means to achieve better abilities. Psychiatrists generally agreed on the need for more extensive cooperation and that the GPs could treat mild and moderate disorders provided that they could, when needed, receive advice and guidance from specialists. Both GPs and specialists emphasised a need for clear guidelines on the division of responsibilities and tasks.

A small primary health centre close to Arkhangelsk city with two GPs contributed to the development and testing of the model. In order to gain a better understanding of GPs’ diagnostic skills, all patients who GPs believed were suffering from a mental illness were diagnosed by specialists from APND using M.I.N.I. [26]. Of an adult patient population of about 1000 the GPs had identified only 4.2% with possible mental disorders in contrast to studies from Western countries indicating that around 20–30% of those seeking primary care have significant psychological problems [13]. Specialists’ M.I.N.I. diagnoses showed that among those 42 patients, 15 had organic mental disorders, 10 developmental disabilities, six schizophrenia, five alcoholism, three personality disorders, and three had depressive disorders. These findings strongly indicated that GPs only to a limited degree had identified the most common depression and anxiety disorders among their patients. Discussions between the GPs and the specialists further indicated that GPs’ patients rather presented bodily ailments than psychological problems.

Thus, there was a great need for improved psychiatric diagnostics among GPs. A structured psychiatric interview for general practice (SPIFA) originally developed in Norway [27] was considered the best available diagnostic instrument. It was translated into Russian and training in its use was carried out in Arkhangelsk. Its utility was tested in 2011 in a study where five GPs used SPIFA on 50 consecutively consenting patients over the age of 18 in their practice (n = 250) while one specialist diagnosed every fifth of those using both SPIFA and M.I.N.I. (n = 50). About 90% of the patients who were invited to participate in the SPIFA interviews consented. The prevalence rates of the most frequent disorders (total 36.8%: depression 8.8%, generalised anxiety disorder 7.6%, social phobia 5.2%, alcohol dependency 4.4%, somatoform disorder 4.0%, adjustment disorder 4.0, agoraphobia 2.8%) were comparable to previous studies in primary care in Western countries [13]. There was good agreement between the specialist’s SPIFA and M.I.N.I. scores for the most frequent disorders (mean kappa 0.87; range 0.34–1.0) and the agreement between GP and specialist SPIFA scores for the same disorders was also acceptable (mean kappa 0.58; range 0.47–0.79) and comparable with a Norwegian study where the mean kappa value was 0.71 [27]. Although the study has limitations related to a low number of patients and low base rate of several of the diagnosed conditions, it may nevertheless be indicative of the SPIFA instrument being useful in our context.

The mean duration of the GPs’ SPIFA interviews was 18 min for the screening part and 25 min for the manual part. The GPs expressed a strong need for a tool like SPIFA and all expressed a need for more training. At the end of the SPIFA interviews, almost all patients expressed a high degree of satisfaction with the interview.

To improve GPs’ access to specialist advice and guidance in areas with large geographic distances between professionals, such as Arkhangelsk County where only 1.2 million inhabitants live in an area of about 600,000 km^2^, use of ICT based communication is particularly important. An ICT-based guidance centre was recently established at APND and professionals were trained in consultation/supervision skills.

### A model for systematic cooperation between GPs and specialists: the Pomor model in psychiatry

Based on this appraisal, the Pomor model was developed from 2011 in collaboration between APND, a primary health care centre close to Arkhangelsk, a group of GPs, and the Russian–Norwegian project group.

#### Assessment of patients’ treatment needs and distribution of treatment responsibilities

General practitioners assess all patients’ mental health condition. In order to estimate different patients’ needs for mental health treatment and care from GPs and specialists, two groups of patients were examined and evaluated both by specialists and GPs: (a) a representative group of patients from APND and (b) patients in primary care with an identified mental disorder. Three categories of patients were identified:

1. Patients with severe mental disorders with a need for active specialist treatment (around 1/5 of the patients diagnosed with a mental disorder). When needed, the specialists consult the GPs concerning patients’ family relations and social issues.
2. Patients with moderate mental disorders (around 1/2) where adequate treatment can be provided by a GP in combination with joint consultation with specialists when needed.
3. Patients in stable remission following specialised treatment and patients with mild depression (around 1/3) who can primarily be treated and followed up by a GP, in combination with specialist consultations if necessary.

In addition, GPs should be informed as soon as possible about which patients need following up after completion of specialist treatment.

Once a year, GPs and specialists from APND have a joint review of GPs patients with diagnosed mental disorders concerning diagnoses, treatment, cooperation, and distribution of treatment responsibilities.

#### ICT-based collaboration methods within the model

An essential prerequisite for being able to attend to these tasks (i) with the necessary expertise is systematic use of the ICT-based guidance centre at APND.

Cooperation takes place through face-to-face/video meetings with patient, GP and specialist present—allowing for shared decision-making on diagnosis, treatment goals and treatment methods as well as face-to-face and video/telephone consultations between GPs and a specialist.

If necessary, the treatment responsibility for psychiatric patients is redistributed between GPs and specialists.

Consultative support and advice from specialists increase the competence and motivation of the GPs and improve the quality of care for patients with mental health problems.

#### Support and further develop GPs’ psychiatric diagnostic and treatment skills

The institutionalised collaboration between GPs and specialists on clinical issues provides continuous competence building. In addition, the GPs were given specific training in psychiatric diagnostic skills (SPIFA) and in conversation skills focusing the importance of good personal contact, clarification of the problem in a psychosocial context and on the use of mainly cognitive techniques. Four yearly 3-day seminars were held for 4 years. Based on the experiences of these seminars, where 10 GPs and four specialists participated, manuals for psychiatric diagnostics and conversation therapy for GPs have been prepared. Training also took place and through teaching practices at APND.

The main elements of the Pomor model: training in psychiatric diagnostics and conversation therapy and systematic collaboration with specialists, as well as teaching practices at APND and other psychiatric institutions, have now been included in a 36 h compulsory family doctor training program with NSMU as the responsible institution.

##### Initial evaluation of the model

At the initiation of the model and a half years later, the chief physician at the participating primary health centre (NR) carried out structured individual interviews with the 42 patients who initially had identified mental disorders. Everyone expressed the need for professional follow-up of their ailments, everyone wished that this should be taken care of by their GPs and everyone so positive that, when needed, there was a collaboration between GP and specialists.

Patient feedback during the whole period show high satisfaction with the treatment and care of the GPs, both among those with moderate and mild mental disorders as well as those being followed up by GPs following discontinuation of treatment in the specialist health service. The patients also convey that the experienced level of stigma is lower when meeting their GP than when meeting a specialist at a specialised clinic. They are also positive towards the cooperation between the GPs and the specialists, including being positive towards joint consultations with both GPs and a specialist present.

The GPs ‘and specialists’ evaluation of the model has been expressed in conjunction with the regular annual cooperation meetings between the GPs and the specialists and through ongoing patient-related cooperation. While the GPs were initially sceptical about the idea that their patients would talk with them about mental health issues at all, most patients were highly motivated to talk about their mental health and the GPs were generally very pleased that they had managed to meet, understand and help their psychiatric patients much more effectively than they had expected. Primarily, they emphasised the importance of specialist assistance having been available when they needed it. Although GPs did not always make use of specialist assistance, the certainty that this support was available was of great importance in ensuring that they generally took responsibility for the treatment of their assigned patients. The importance of the training provided in psychiatric diagnostics was also emphasised.

The participating specialists were initially sceptical of whether GPs had the necessary professional and experimental prerequisites to provide good mental health care under their guidance. They were therefore surprised by the quality of the GPs’ work, which was also confirmed by talks with the patients.

While only 42 patients with mental disorders were identified in 2011, the number was 70 in 2018. All increases relate to anxiety and depression disorders. In addition, the hospitalisation rates from this district to the regional psychiatric hospital decreased, probably due to better continuity of care following discharge from the hospital.

### Implementation and dissemination of the model

Arkhangelsk neuropsychiatric dispenser, as part of its obligations, takes the initiative to implement the model in collaboration with the selected primary health care centres and routines for collaboration on clinical issues and training are incorporated into the routines of the cooperating units. The positive experiences in the test district have led to the model now being implemented in half of county districts and it has been decided that the entire county should eventually be included. Experiences with the model has also been requested in other parts of the Russian Federation.

Further development of the model is based on ongoing systematic evaluation of experiences with the model based on patient data from all participating districts (symptoms, level of functioning and treatment satisfaction), staff satisfaction scores as well as journal and register data from participating units.

## Discussion

A key element of the model is the creation of an ICT-based competence network including the involved primary care centres and APND, enabling GPs to have access to specialist assistance when needed in their work with their psychiatric patients. Another key element is that the GPs assess the mental health condition of all their patients and that treatment responsibility for patients in need of mental health care is distributed between GPs and specialists according to the severity of the problems. We do not know of other cooperation models where the treatment responsibility for psychiatric patients in primary care is systematically distributed between GPs and specialists. The Norwegian DPS model [10] enables such practices but we are currently not familiar with them.

The fact that APND has a defined responsibility for implementing the model in collaboration with the primary care centres involved, motivates the primary health service to systematically work with the patients’ mental health problems. The active role of specialised community mental health centres (APND) in the implementation of the model separates it from other known models [10].

In areas with large geographical distances between local health providers and available specialists, such as in Arkhangelsk County and Northern Norway, the advantages of ICT-based networking possibilities are increasing. Organised competence networks, face-to-face and via ICT systems, provide primary health care professionals with access to specialists’ advice and help when needed, and seem to generate competence and coping beyond what the individual GP is typically trained for, as assessed by the GPs themselves and the collaborative specialists.

Organised training in psychiatric diagnostics and conversation skills, as well as the teaching practices at APND, ensures basic professional competence in the GPs.

In addition, the 36-h psychiatry postgraduate training module emphasising the Pomor model, now in place at the Northern State Medical University in Arkhangelsk (NSMU), ensures that all GPs get to know the model as part of their specialist training.

During the work with the model, we encountered various obstacles. Cooperating GPs did not initially believe that their patients would let themselves be interviewed about their mental health. However, on the contrary, patients unequivocally expressed that this was something they liked and had missed. Some cooperating specialists also initially expressed scepticism as to whether GPs actually had the prerequisites for understanding and helping psychiatric patients in a competent way. This scepticism also shrank as the specialists gained insight into GPs’ work with the patients through the systematic collaboration. We encountered few structural or institutional obstacles after the model was presented and discussed thoroughly both by managers and employees. It appeared that many professionals in primary care had long been frustrated that they had not been able to provide adequate care to people with obvious mental health problems. This experience harmonised with the patients’ appreciating being met by doctors who showed interest in their mental health condition, and their appreciation that it was less stigmatising to talk with GPs than with specialists about mental health issues.

In our efforts to integrate mental health work into primary health care in Arkhangelsk County, we rarely encountered such obstacles as a narrow bio-medical model mainly focusing on medical treatment and prognostic and therapeutic pessimism as previously described in the Sverdlovsk study [21]. However, our partners, both treatment staff and managers, were selected based on their interest in the project’s goals and may not be representative of their respective groups in the whole county. As the model is currently being implemented in large parts of the county, we may expect bigger obstacles. However, there is reason to assume that the institutional approach where APND actively takes initiative and offers its services to the district health centres strongly motivates to include psychiatry in primary care services with a corresponding reduction in defensive attitudes and arguments.

The preconditions of success with our initiative were complex and related to evidence, contextual factors and facilitation [28]. Extensive evidence confirms that mental health problems are common and represent serious public health challenges [2–9], and that the identification and treatment of mental disorders must be made available in primary health care in order to meet the need for help [2, 13, 21]. Local data systematically collected from GPs, specialists and patients helped to illuminate local conditions that limited and enabled mental health work in primary health care.

A basic prerequisite for the development and realisation of the Pomor model is that it complies with federal and regional health policy guidelines that mental health care should be integrated into primary care. It has also been important that the key-persons in the development, testing, and implementation of the model were both members of the project group and in leading positions in the involved health services, and thus had in-depth knowledge of local needs and resources and the necessary authority to implement the model. As the work progressed, Norwegian economic and professional contributions gradually declined while the Russian efforts became correspondingly larger. The management of the program’s implementation and dissemination now rests fully with the Russian authorities and institutions involved.

The development and implementation of the model has been facilitated by good and stable relationships and emphasis on mutual understanding and respect for each other’s prerequisites and experiences. Good translation between languages and cultures both in informal and formal settings has been largely taken care of by a member of the project group (GR) who has professional networks in both countries, speaks both languages fluently and has in-depth knowledge of the culture and psychiatry of both countries. Conflicting expectations and misunderstandings have thus been reduced and easier to correct. In addition, Russian project team members in charge of APND (VY) and the initial testing of the model in one primary health care centre (NR) have ensured that their employees have been motivated and engaged in the work of implementing the model.

## Conclusions

Our Russian–Norwegian cooperation has pioneered a model for the integration of mental health into primary care in a geographic area with large geographic distances between health care centres. Competence networks that give GPs access to specialist assistance when they need it, the distribution of responsibility for the treatment of people with mental disorders between GPs and specialists, and a specialist health service that has a defined responsibility for ensuring that the model is realised, and systematic diagnostic skills training, seem to be of crucial importance to the success of the model. It appears to be well adapted to local conditions and is sustainable. We also assume that the model could inspire the integration of mental health into primary care in other contexts.
